# Edward VII

**Published:** 1910-06

**Authors:** 


**A Monthly Review of Medicine and Surgery.**

*EDITOR*

WILLIAM WARREN POTTER, M. D.

All communications, whether of a literary or business nature, books for review and xohanges, should be addressed to the editor, 238 DELAWARE AVENUE, BUFFALO, N.Y.

VOL. LXV. JUNE, 1910. No. 11

### Edward VII

THE death of King Edward, which occurred May 6, 1910, was a shock to the peoples of the civilised world, and to none more than to citizens of the United States. A strong attachment has grown up of late between Americans and the British people, fostered and greatly strengthened by the tactful conduct of King Edward, during the nine years of his reign. He was not only a true friend of the United States as a nation, but he stood for the same principles of peace and progress that characterise the thought and action of citizens of this republic.

It is not within our province to eulogise the life and character of the great English king, just passed away, but it is not inopportune for us to remark,—and it is entirely within the boundaries of truth for us to affirm,—that in Edward VII. were embodied the combined elements of a great ruler, a great citizen, and an honest man. Moreover, he was perhaps the ablest representative of monarchial power of his time; certainly he had no superior. He was a born diplomat, had great knowledge of men, was skilled in statecraft, and possessed an amiability of temper that fitted him to deal with the great questions of government. He was every inch a king, and his rule was all too short!

As to the cause of his majesty's illness and death, a justifiable reticence was maintained during his lifetime by the physicians in attendance. But now that all is over it is pertinent to discuss these questions, with a view to determine the facts. The *British Medical Journal* seems to present the case with authoritative frankness. We quote freely from its edition of May 16, in which it makes a summary of King Edward's illness. It says:

For years the king suffered from emphysema and a tendency more or less acute to bronchitis, with the usual symptoms of a distressing, ineffective cough and difficulty of breathing. There was crepitation at the bases of both lungs, indicating a chronic impediment to the free passage of air in the smaller bronchial tubes. He was subject to attacks of laryngitis, which produced slight spasms of the vocal chords, but except for some inflammatory thickening at the hinder part of the glottis and chronic catarrh of the throat, there was no trace of disease in the upper air passages.

The king, in short, had what is known as smoker's throat. This and the congestion and thickening due to this cause, combined with the loss of elasticity in the lungs, made it increasingly difficult for him to clear his chest. The strain thrown upon the heart by the obstruction to the passage of blood through the lungs, caused by the collection of secretion in the bronchial tubes, had its natural sequel in the dilation of the right ventricle, and the natural cause of death was heart failure due to increasing difficulty in the pulmonary circulation.

In short, it was a case of a type to be seen every day in thousands of elderly persons. The cause of death in such cases is purely mechanical, the overlain heart being stopped by the increasing resistance in the lungs. Could the king have been induced to spare himself more he probably would have lived many years longer. He had, indeed, suffered from glycosuria of a varying degree for a long time, but this does not, so far as can be judged, tend to shorten life.

Another condition which must have caused considerable discussion at times was a certain weakness in the abdominal wall at the site of the operation for appendicitis which was performed in 1902.

After referring to the king's illness at Biarritz and the fact he felt obliged to return home to play his part in the constitutional crisis, the *Journal* continues:

The hurried journey would have taxed his strength, even had he been in perfect health. Suffering as he was from the effects of a recent illness, the king might fairly have been excused from facing the risk of returning from the south to the cloudy skies, cold winds and showers of the treacherous English spring. The result might almost have been foretold. Though the end came with startling suddenness to his people, it was clear to those about him that the end was imminent before any cause for alarm spread outside the palace. The examination on Thursday revealed the real nature of the situation to those who could read between the lines.

The funeral of the king was a splendid pageant, a deserved tribute to the memory of a great sovereign, one that perhaps only England could pay with such magnificent splendor. It was a dignified and solemn mourning cortege from Westminster Hall to Saint George's Chapel, made up of nine crowned heads, queens and potentates, soldiers of renown from every country in Europe, a sorrowing household from the queen mother to ladies in waiting, special ambassadors, among whom was Theodore Roosevelt, representing the United States, distinguished men in public life, —all following the body of the king through a lane of soldiery three miles long, stretching from Westminster to Paddington, all under command of the Duke of Norfolk, as Earl Marshal. A pathetic picture was presented in the leading of the king's charger and his favorite terrier behind the gun carriage that bore the remains. Hundreds of thousands of citizens flanked the column on either side, and when Windsor was reached a solemn church service was read according to the established rules, the ritual of the Order of the Garter was repeated, and then the body was deposited in the crypt.

